# Evaluation of elevated soybean meal diets with inclusion of exogenous enzymes on the effect of growth performance and digestibility

**DOI:** 10.1093/tas/txag070

**Published:** 2026-05-20

**Authors:** Caitlyn M Phillips, Dalton C Humphrey, Steven L Trabue, Jon R Bergstrom, Laura L Greiner

**Affiliations:** Department of Animal Science, Iowa State University, Ames, IA, 50011, United States; Department of Animal Science, Iowa State University, Ames, IA, 50011, United States; USDA- ARS National Laboratory for Agriculture and the Environment, Ames, IA, 50011, United States; Animal Nutrition & Health, DSM-Firmenich, Plainsboro, NJ, 08536, United States; Department of Animal Science, Iowa State University, Ames, IA, 50011, United States

**Keywords:** enzymes, nitrogen, phosphorus, soybean meal, sulfur, swine

## Abstract

Forty-eight crossbred gilts (52.55 ± 4.12 kg) were used to evaluate the effect of high soybean meal (SBM) inclusion with dietary enzymes on growth and nutrient balance. Two sets of 24 gilts were placed in metabolism stalls and randomly assigned to one of six dietary treatments (8 pigs/trt). Experimental diets were fed in two, 12-d phases, urine and feces were collected for 72 h to assess nutrient digestibility and retention. Diets included: (i) negative control diet [NC, corn-soy (22% SBM) + synthetic AA], (ii) positive control diet, (PC, 41% SBM replacing synthetic lysine), (iii) PC + xylanase (WX), (iv) PC + multi-enzyme (VP, hemicellulase and pectinase), (v) PC + WX + VP (WXVP), and (vi) PC + 1500 FYT/kg phytase (SD). All but SD contained 600 FYT/kg phytase. Data were analyzed as repeated measures in SAS 9.4 (Statistical Analysis System, Cary, NC) with treatment, phase, and block as fixed effects and pig served as the experimental unit. Growth did not differ across treatments (*P* ≥ 0.13). Apparent total tract digestibility (ATTD) of DM and GE were unaffected (*P* ≥ 0.10), ash ATTD increased in high SBM diets (*P* < 0.01). Intake of nitrogen (N), phosphorus (P), calcium (Ca), and sulfur (S) increased with high SBM (HSBM) diets compared to NC (*P* < 0.01). Digestibility of nitrogen (N), phosphorus (P), and sulfur (S) increased in pigs fed HSBM diets (*P* < 0.01), while calcium (Ca) digestibility and retention were greatest in the PC and elevated phytase diets compared to the NC (*P* < 0.01). Absolute retention of N, P, and S was greater in higher SBM diets (*P* ≤ 0.02), though enzyme inclusion had minimal effects. Diet WX was intermediate with other HSBM treatments and NC for N retention (*P* < 0.01), WXVP was intermediate with other HSBM treatments and NC for P and S retention (*P* ≤ 0.02). Total N excretion was greater in WX, VP, and WXVP vs NC (*P* = 0.01), while enzyme supplemented diets did not differ from each other, PC and SD showed intermediate excretion levels similar to NC. Intake and digestibility of S and P increased with higher SBM, without increasing S and P excretion (*P* ≥ 0.12). Total Ca excretion was greater in WXVP vs VP (*P* = 0.03). Digestible and metabolizable energy values were similar across treatments (*P* ≥ 0.22). In conclusion, increasing SBM by 19% did not affect weight gain but increased intake and retention of N, P, Ca, and S, while dietary enzyme inclusion provided limited benefits in corn-soy diets, except for SD, which significantly improved Ca retention.

## Teaser

Feeding high levels of soybean meal with combination of dietary enzymes in swine diets did not negatively impact pig weight gain. Elevated soybean meal diets enhanced absolute nitrogen, phosphorus, calcium, and sulfur retention in growing pigs, while dietary enzyme inclusion offered limited benefits in corn-soy diets.

## Graphical Abstract

**Graphical Abstract:**
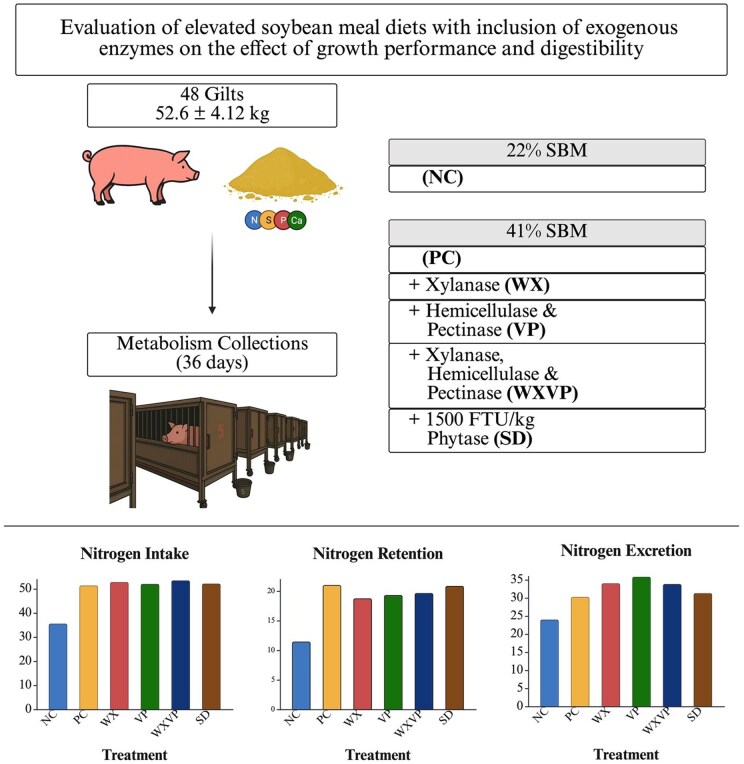

## Introduction

Soybean meal (SBM) is commonly used in swine diets due to its amino acid (AA) profile, providing a quality protein source and geographic proximity to swine production. Crystalline AA and cereal grains or their by-products are typically utilized in late-finishing diets to minimize feed costs and nitrogen excretion. With increased availability of crystalline AA, swine diets have become more precise to the pig’s nutrient recommendations. The swine industry typically formulates diets to meet the best-cost formulation, which depends on the crystalline AA and SBM market prices. It has been reported that a negative impact on growth performance from 30% SBM inclusion in finishing pigs may be observed due to SBM antinutritional factors and potential imbalances of other nutrients (Woyengo et al. 2017). However, a recent study has reported that finishing pig diets consisting of 48% SBM did not impact growth performance, but increased N excretion (Swanstrom et al. 2023).

Higher SBM inclusion in swine diets will result in elevated crude protein (CP) and have further effects on nitrogen (N) excretion. Each 1% CP fluctuation yields a linear 8% change in N excretion levels, regardless of bodyweight (Shurson and Kerr 2023). Manure N, phosphorus (P), and sulfur (S) contribute towards ammonia, nitrate, hydrogen sulfide, and other odorous and environmental concerns (Hatfield 2002). This rise in environmental concern has led to research investigating the effects of diet on downstream environmental impacts (Trabue et al. 2011).

Two primary antinutritional factors (ANF) present in corn-SBM based diets that limit nutrient utilization are phytate and non-starch polysaccharides (NSP). Phytate binds P, Ca, Zn, Na, AA, and carbohydrates, reducing bioavailability (Dersjant-Li et al. 2015). Soluble NSPs have two modes of action in relation to antinutritional factors; they bind nutrients and increase digesta viscosity, which reduces absorption rates (Ward 2021). Diet formulation in swine production requires consideration of ANF and possibilities for release of bound nutrients from seed and cereal grain ingredients. Phytase inclusion is a common practice in swine nutrition to enhance P digestibility and reduce P excretion (Cromwell et al. 1995). When phytase is “super-dosed” (supplemented greater than 3 times the standard release curve), an additional release of minerals and sometimes improvements in growth have been observed (Humer et al. 2015).

Carbohydrases, including xylanase, β-glucanase, pectinase, and hemicellulase, cleave various NSP matrices in swine diets. Their efficacy is well established in poultry, where reductions in digesta viscosity have also yielded improved feed efficiency (Adeola and Bedford 2004). Torres-Pitarch et al. (2019) have summarized the efficacy of carbohydrases varies by diet composition, showing more consistent results when multi-enzyme complexes are matched to fiber type. Insoluble pectins and xyloglucans are present in corn-SBM diets and appear less responsive than wheat- or barley-based formulas with exogenous carbohydrase inclusion (Bach-Knudsen 2014; Villca et al. 2016; Wang et al. 2018).

The literature suggests that dietary enzymes can mitigate specific antinutritional factors, yet their activity on high-SBM and corn-based diets remains undetermined. The objective of this study was to test whether individual or combinations of carbohydrases, enhance nutrient digestibility and N retention without compromising growth performance in growing pigs fed corn-soybean based diets with HSBM inclusion. The authors hypothesize that supplementing phytase, xylanase, and a hemicellulase and pectinase complex will improve the nutrient digestibility and retention in pigs fed diets containing HSBM.

## Materials and methods

All experimental protocols were in correspondence to the ethical and humane use of animals for research according to the Guide for the Care and Use of Agricultural Animals in Research and Teaching (FASS, 2010) and were approved by the Institutional Animal Care and Use Committee at Iowa State University (IACUC 23-003).

### Animals and experimental design

This experiment was completed at the Iowa State University Swine Nutrition Farm (Ames, IA), utilizing a total of 48 crossbred gilts (PIC 337 × 1050, Genus, Hendersonville, TN) with an initial body weight (BW) of 52.55 ± 4.12 kg. This study consisted of two replicates of 24 gilts. Pigs had originated from a porcine reproductive and respiratory syndrome virus (PRRSV) vaccinated herd and had no other clinical signs of disease. All pigs were observed and evaluated twice daily in metabolism stalls for signs of lameness, lethargy, and illness.

On D0, pigs were weighed and placed in metabolism stalls (0.7 × 1.7 m) equipped with a slatted floor, stainless steel feeder, and nipple waterer. The metabolism stalls were in a temperature-controlled room at a temperature of approximately 21 °C. Pigs were blocked by initial BW and randomly assigned within a block to one of six dietary treatments.

### Diets and feeding

Pigs were fed one of six dietary treatments; (i) a basal control diet (NC) formulated to represent a standard commercial diet with AA inclusions and 22% to 15.6% SBM, (ii) high soybean meal (HSBM) diet (PC) formulated to replace all synthetic lysine, (iii) HSBM diet with xylanase (Ronozyme WX 5000, DSM) (WX), (iv) HSBM diet with inclusion of a multi-enzyme additive including hemicellulase and pectinase (Ronozyme VP, DSM) (VP), (v) HSBM diet with inclusion of xylanase (Ronozyme WX 5000, DSM) and a multi-enzyme additive including hemicellulase and pectinase (Ronozyme VP, DSM) (WXVP), and (vi) HSBM diet with 1500 phytase units (FYT) per kg of phytase (HiPhorious, DSM) (SD). Phytase (HiPhorious, DSM Nutritional Products, Parsippany, NJ) was included in all diets excluding the SD diet to provide 600 phytase units (FYT) per kg of diet and was assumed to release 0.15% available phosphorus. The SD diet contained 1500 FYT/kg and was assumed to release 0.21% available phosphorus. Dietary enzymes were analyzed by DSM-Firmenich Technical Marketing Analytical Services Team for enzymatic activity.

All pigs were fed a common diet during phase 1 (Table 1) from day 0 to 12 for baseline measurements. Following baseline collections, experimental diets were fed in two dietary phases, with phase 2 diets (Table 2) being fed from d 12 to d 24 and phase 3 diets (Table 3) fed from d 24 to d 36. Standardized ileal digestible (SID) lysine to metabolizable energy (ME) ratios of diets containing HSBM maintained consistent in all diet phases. All diets were formulated to meet or exceed NRC (2012) recommendations for vitamins, minerals, and amino acids. Daily feed allowance was established during the acclimation period of each phase and applied uniformly across treatments to ensure comparable intake and energy levels across treatments. Diets were formulated to be isocaloric within diet phase on an ME basis. Lower lysine levels were utilized in the NC diet to induce a change in nitrogen excretion. Dietary enzymes were included according to the ingredient specifications and label information provided by the manufacturer.

**Table 1:** Ingredients and nutrient composition of common diet fed to growing pigs (53–62 kg) for baseline measurements of nutrient excretion (as-fed basis).

Ingredient, %	Common diet^e^
** Corn**	71.000
** Soybean meal**	26.650
** Monocalcium phosphate**	0.232
** Calcium carbonate**	0.889
** Sodium chloride**	0.500
** l-lysine HCl**	0.214
** l-threonine**	0.102
** dl-methionine**	0.092
** Vitamin and mineral premix^a^**	0.300
** Phytase^b^**	0.025
**Calculated composition**	
** Metabolizable energy, Mcal/kg**	3.34
** Crude protein, %**	17.99
** Calcium, %**	0.50
** Phosphorus, %**	0.41
** Available phosphorus, %**	0.27
** Ca: P**	1.24
** Ca: AvP^c^**	1.90
** Total Lys, %**	1.10
** SID^d^ Lys, %**	0.98
** SID Thr: Lys**	0.65
** SID Met + Cys: Lys**	0.58
** SID Trp: Lys**	0.19
** SID Val: Lys**	0.69
** SID Leu: Lys**	1.32
** SID Ile: Lys**	0.63
**Analyzed composition**	
** Gross energy, Mcal/kg**	3.85
** Dry matter, %**	88.94
** Ash, %**	3.96
** Crude protein, %**	15.54
** Total Lys, %**	1.10
** Total Cys, %**	0.27
** Total Met, %**	0.38
** Calcium, %**	0.67
** Phosphorus, %**	0.42
** Ca: P**	1.60
** Sulfur, %**	0.19

a Vitamin and mineral premix; Provided 4594-IU vitamin A, 525-IU vitamin D, 37.5-IU vitamin E, 2.25-mg vitamin K, 8.25-mg riboflavin, 42-mg niacin, 20.25-mg pantothenic acid, 0.04-mg vitamin B12, 12-mg Cu (copper sulfate), 0.28-mg I (potassium iodate), 160-mg Fe (ferrous sulfate), 0.30-mg Se (sodium selenate), and 160-mg Zn (zinc sulfate) per kg of the diet.

b Phytase; phytase activity provided 600 FYT per kg diet. HiPhorious, (DSM Nutritional Products, Parsippany, NJ); provided 600 FYT/kg, assuming 0.15% available phosphorus release.

c Ca: AvP; Calcium to available phosphorus ratio.

d SID; standardized ileal digestible.

e Common Diet; All pigs were fed a common diet during Phase 1from day 0 to 11 for baseline measurements.

**Table 2:** Ingredients and nutrient composition of phase 2 dietary treatments (NC, PC, WX, VP, WXVP, SD) (as-fed basis) to growing pigs (62–75 kg) to evaluate the effect of elevated soybean meal (41%) and exogenous enzymes on nutrient excretion.

Ingredient, %	Dietary treatment					
	**NC** ^g^	**PC** ^h^	**WX** ^i^	**VP** ^j^	**WXVP** ^k^	**SD** ^l^
** Corn**	75.740	57.025	57.025	56.990	56.990	57.025
** Soybean meal**	22.000	41.000	41.000	41.000	41.000	41.000
** Monocalcium phosphate**	0.260	0.181	0.179	0.178	0.178	0.150
** Calcium carbonate**	0.840	0.868	0.867	0.866	0.866	0.860
** Sodium chloride**	0.500	0.500	0.500	0.500	0.500	0.500
** l-lysine HCl**	0.192	–	–	–	–	–
** l-threonine**	0.079	0.026	0.026	0.026	0.026	0.026
** dl-methionine**	0.065	0.075	0.075	0.075	0.075	0.075
** Vitamin and mineral premix^a^**	0.300	0.300	0.300	0.300	0.300	0.300
** Soybean oil**	–	–	–	0.011	0.013	–
** Phytase^b^**	0.025	0.025	0.025	0.025	0.025	0.063
** Xylanase^c^**	–	–	0.003	–	0.003	–
** Multicomponent enzyme^d^**	–	–	–	0.025	0.025	–
**Calculated composition**						
** Metabolizable energy, Mcal/kg**	3.34	3.33	3.33	3.33	3.33	3.33
** Crude protein, %**	16.11	23.37	23.37	23.37	23.37	23.37
** Calcium, %**	0.64	0.71	0.71	0.71	0.71	0.71
** Phosphorus, %**	0.40	0.45	0.45	0.45	0.45	0.44
** Available phosphorus, %**	0.27	0.27	0.27	0.27	0.27	0.27
** Ca: P**	1.62	1.56	1.56	1.56	1.56	1.61
** Ca: AvP^e^**	2.38	2.60	2.60	2.60	2.60	2.60
** Total Lys, %**	0.96	1.32	1.32	1.32	1.32	1.32
** SID^f^ Lys, %**	0.85	1.15	1.15	1.15	1.15	1.15
** SID Thr: Lys**	0.37	0.48	0.48	0.48	0.48	0.48
** SID Met + Cys: Lys**	0.59	0.58	0.58	0.58	0.58	0.58
** SID Trp: Lys**	0.20	0.22	0.22	0.22	0.22	0.22
** SID Val: Lys**	0.71	0.78	0.78	0.78	0.78	0.78
** SID Leu: Lys**	1.40	1.42	1.42	1.42	1.42	1.42
** SID Ile: Lys**	0.64	0.74	0.74	0.74	0.74	0.74
**Analyzed composition**						
** Gross energy, Mcal/kg**	3.86	3.89	3.91	3.93	3.90	3.91
** Dry matter, %**	88.97	89.25	88.54	88.64	89.47	89.22
** Ash, %**	3.75	4.50	4.42	4.59	4.51	4.29
** Crude protein, %**	15.06	20.65	21.88	22.77	21.51	21.43
** Total Lys, %**	0.94	1.29	1.32	1.29	1.25	1.27
** Total Cys, %**	0.26	0.33	0.34	0.35	0.34	0.34
** Total Met, %**	0.30	0.38	0.41	0.38	0.38	0.42
** Calcium, %**	0.61	0.82	0.67	0.69	0.72	0.71
** Phosphorus, %**	0.44	0.48	0.49	0.51	0.47	0.47
** Ca: P**	1.38	1.71	1.38	1.37	1.52	1.51
** Sulfur, %**	0.18	0.24	0.23	0.24	0.23	0.23

a Vitamin and mineral premix; provided 4594-IU vitamin A, 525-IU vitamin D, 37.5-IU vitamin E, 2.25-mg vitamin K, 8.25-mg riboflavin, 42-mg niacin, 20.25-mg pantothenic acid, 0.04-mg vitamin B12, 12-mg Cu (copper sulfate), 0.28-mg I (potassium iodate), 160-mg Fe (ferrous sulfate), 0.30-mg Se (sodium selenate), and 160-mg Zn (zinc sulfate) per kg of the diet.

b Phytase; phytase activity provided 600 FYT per kg diet. HiPhorious, (DSM Nutritional Products, Parsippany, NJ); provided 600 FYT/kg, assuming 0.15% available phosphorus release. Super-dose diet provided 1500 FYT/kg, assuming 0.21% available phosphorus release.

c Xylanase, Ronozyme WX 5000 (DSM Nutritional Products).

d Multicomponent enzyme, Ronozyme VP (DSM Nutritional Products).

e Ca: AvP; Calcium to available phosphorus ratio.

f SID, standardized ileal digestible.

g NC; basal control diet formulated to represent a standard commercial diet with AA inclusions and 22% SBM.

h PC; high soybean meal diet (41%) formulated to replace all synthetic lysine.

i WX; high soybean meal diet (41%) with xylanase (Ronozyme WX 5000, DSM-Firmenich).

j VP; high soybean meal diet (41%) with inclusion of a multi-enzyme additive including hemicellulase and pectinase (Ronozyme VP, DSM- Firmenich).

k WXVP; high soybean meal diet (41%) with inclusion of xylanase (Ronozyme WX 5000, DSM-Firmenich) and a multi-enzyme additive including hemicellulase and pectinase (Ronozyme VP, DSM-Firmenich).

l SD; high soybean meal diet (41%) with 1500 phytase units (FYT) per kg of phytase (HiPhorious, DSM-Firmenich).

**Table 3:** Ingredients and nutrient composition of phase 3 dietary treatments (NC, PC, WX, VP, WXVP, SD) (as-fed basis) to growing pigs (75–87 kg) to evaluate the effect of elevated soybean meal (35%) and exogenous enzymes on nutrient excretion.

Ingredient, %	Dietary treatment					
	NC^g^	PC^h^	WX^i^	VP^j^	WXVP^k^	SD^l^
** Corn**	81.850	63.050	63.092	63.092	63.092	63.092
** Soybean meal**	15.64	35.000	35.000	35.000	35.000	35.000
** Monocalcium phosphate**	0.187	0.173	0.158	0.158	0.158	0.081
** Calcium carbonate**	0.971	0.886	0.870	0.848	0.845	0.914
** Sodium chloride**	0.500	0.500	0.500	0.500	0.500	0.500
** l-lysine HCl**	0.300	–	–	–	–	–
** l-threonine**	0.119	0.011	0.011	0.011	0.011	0.011
** dl-methionine**	0.068	0.041	0.041	0.041	0.041	0.041
** l-valine**	0.026	–	–	–	–	–
** l-tryptophan**	0.015	–	–	–	–	–
** Vitamin and mineral premix^a^**	0.300	0.300	0.300	0.300	0.300	0.300
** Soybean oil**	–	0.015	–	–	–	–
** Phytase^b^**	0.025	0.025	0.025	0.025	0.025	0.0625
** Xylanase^c^**	–	–	0.003	–	0.003	–
** Multicomponent enzyme^d^**	–	–	–	0.025	0.025	–
**Calculated composition**						
** Metabolizable energy, Mcal/kg**	3.35	3.34	3.34	3.34	3.34	3.34
** Crude Protein, %**	13.77	20.99	20.99	20.99	20.99	20.99
** Calcium, %**	0.65	0.69	0.68	0.68	0.68	0.68
** Phosphorus, %**	0.35	0.43	0.43	0.43	0.43	0.41
** Available phosphorus, %**	0.26	0.26	0.26	0.26	0.26	0.26
** Ca: P**	1.85	1.62	1.62	1.62	1.62	1.65
** Ca: AvP^e^**	2.50	2.62	2.62	2.62	2.62	2.62
** Total Lys, %**	0.88	1.16	1.16	1.16	1.16	1.16
** SID^f^ Lys, %**	0.78	1.01	1.01	1.01	1.01	1.01
** SID Thr: Lys**	0.65	0.65	0.65	0.65	0.65	0.65
** SID Met + Cys: Lys**	0.58	0.58	0.58	0.58	0.58	0.58
** SID Trp: Lys**	0.18	0.22	0.22	0.22	0.22	0.22
** SID Val: Lys**	0.68	0.80	0.80	0.80	0.80	0.80
** SID Leu: Lys**	1.33	1.48	1.48	1.48	1.48	1.48
** SID Ile: Lys**	0.56	0.75	0.75	0.75	0.75	0.75
**Analyzed composition**						
** Gross energy, Mcal/kg**	3.75	3.87	3.85	3.87	3.88	3.86
** Dry matter, %**	86.65	87.28	87.86	87.89	87.76	87.84
** Ash, %**	3.04	4.11	4.42	4.24	4.13	4.14
** Crude Protein, %**	12.53	19.68	18.94	18.61	19.06	18.89
** Total Lys, %**	0.78	1.06	1.13	1.11	1.01	1.12
** Total Cys, %**	0.21	0.29	0.32	0.30	0.28	0.32
** Total Met, %**	0.29	0.37	0.35	0.32	0.30	0.32
** Calcium, %**	0.58	0.68	0.72	0.66	0.67	0.72
** Phosphorus, %**	0.33	0.45	0.44	0.43	0.41	0.42
** Ca: P**	1.77	1.54	1.64	1.51	1.63	1.73
** Sulfur, %**	0.16	0.20	0.21	0.21	0.20	0.20

a Vitamin and mineral premix; provided 4594-IU vitamin A, 525-IU vitamin D, 37.5-IU vitamin E, 2.25-mg vitamin K, 8.25-mg riboflavin, 42-mg niacin, 20.25-mg pantothenic acid, 0.04-mg vitamin B12, 12-mg Cu (copper sulfate), 0.28-mg I (potassium iodate), 160-mg Fe (ferrous sulfate), 0.30-mg Se (sodium selenate), and 160-mg Zn (zinc sulfate) per kg of the diet.

b Phytase; phytase activity provided 600 FYT per kg diet. HiPhorious, (DSM Nutritional Products, Parsippany, NJ); provided 600 FYT/kg, assuming 0.15% available phosphorus release. Super-dose diet provided 1500 FYT/kg, assuming 0.21% available phosphorus release.

c Xylanase, Ronozyme WX 5000 (DSM Nutritional Products).

d Multicomponent enzyme (hemicellulase + pectinase), Ronozyme VP (DSM Nutritional Products).

e Ca: AvP; Calcium to available phosphorus ratio.

f SID, standardized ileal digestible.

g NC; basal control diet formulated to represent a standard commercial diet with AA inclusions and 15.6% SBM.

h PC; high soybean meal diet formulated to replace all synthetic lysine (35% SBM).

i WX; high soybean meal diet (35%) with xylanase (Ronozyme WX 5000, DSM-Firmenich).

j VP; high soybean meal diet (35%) with inclusion of a multi-enzyme additive including hemicellulase and pectinase (Ronozyme VP, DSM- Firmenich).

k WXVP; high soybean meal diet (35%) with inclusion of xylanase (Ronozyme WX 5000, DSM-Firmenich) and a multi-enzyme additive including hemicellulase and pectinase (Ronozyme VP, DSM-Firmenich).

l SD; high soybean meal diet (35%) with 1500 phytase units (FYT) per kg of phytase (HiPhorious, DSM-Firmenich).

Feed allowance was determined based on the average ad libitum intake during the first acclimation period and set at 2.8 times the maintenance energy requirement (197 kcal × BW^0.6^; NRC 2012) of the average pig BW. Pig BW was collected at the end of each collection period, feed allowance was adjusted based on the new average BW. Feed distribution was split equally into two feedings at 0700 and 1630 h daily. The remaining feed (orts) after 1 h were collected, dried, and weighed. Ad libitum water was provided throughout the entire trial.

### Sample collection

The 36-d experiment was separated into three collection periods, with the common diet (Table 1) being fed during collection period one, phase two experimental diets (Table 2) fed during collection period two, and phase three experimental diets (Table 3) fed during collection period three. Each 12-d collection period was subdivided into two subperiods to facilitate collections of manure gas emissions and nutrient digestibility. Pigs were allowed 6 d of acclimation before collection periods one through three, respectively.

### Apparent total tract digestibility collections

Total quantities of urine and feces were collected twice daily at 0700 and 1630 h and immediately stored at −20 °C. Prior to each collection of urine, 25 mL of 6 N HCl was added to stainless steel buckets to prevent bacterial growth and N volatilization. At the conclusion of each collection, urine was thawed, weighed, and filtered with glass wool (Glass Wool #386060010, Thermo Scientific, Waltham, MA) prior to retaining a subsample and stored again at −20 °C for subsequent analysis. Fecal samples were thawed, weighed, and dried in a convection oven at 75 °C until a constant weight was achieved and were ground to a particle size of 1.0 mm (Wiley Mill 3379-K35, Thomas Scientific, Swedesboro, NJ). Orts were dried in a convection oven at 75 °C until a constant weight was achieved.

### Diet, urine, fecal, and manure analytical methods

Diet and fecal samples were analyzed in duplicate for dry matter (DM) and ash. Diet, feces, and urine were analyzed in duplicate for nitrogen (method 990.03; AOAC 2007; TruMac; LECO Corp., St. Joseph, MI) and gross energy (GE) using a bomb calorimeter (model 6200; Parr Instrument Co., Moline, IL). Benzoic acid (6318 kcal/kg; Parr Instrument Co.) was used as the standard for calibration and was determined to contain 6317.12 ± 9.42 kcal/kg. For urine energy determination, urine was added to 0.50 g of cellulose (Cellulose microcrystalline 50 μm, #9004-34-6; Thermo Scientific, Waltham, MA) and dried at 50 °C for 4 d, 1.5 ml of urine was added on day 0 and 2. A final weight was collected on day 4. Pure cellulose was run in quadruplicate to determine the gross energy value and use as a standard when running dried urine samples and was determined to contain 3951 ± 8.69 kcal/kg. This value was subtracted from the total gross energy value to calculate urine gross energy.

Diet, fecal, urine, and manure were submitted to the USDA-ARS National Laboratory for Agriculture and The Environment (Ames, IA) to be analyzed in duplicate for Ca, P, and S using inductively coupled plasma-mass spectrometry (ICP-OES; Avio 500, Perkin Elmer, Waltham, MA). In brief, approximately one gram (±0.5 g) of sample was acid-digested at 200 °C for 20 min in a microwave (MARSXpress Plus, CEM Corporation) using 10 mL of concentrated nitric acid (Nitric Acid, Optima™, Fisher Scientific, Waltham, MA). Gallium was used as an internal standard to account for any variation in sample introduction between individual samples.

Diet samples were also subject to complete amino acid profiling at the University of Missouri (Columbia, MO) using cation-exchange chromatography coupled with post-column ninhydrin derivatization and quantification (method 982.30 E and 988.15; AOAC 2007).

### Calculations and statistical analysis

Digestible energy (DE) was calculated by subtracting fecal energy from GE intake. Metabolizable energy (ME) was calculated by subtracting urinary energy from DE (CH_4_ losses were omitted). Digestibility and nutrient balance values were calculated using the following equations:


Apparent total tract digestibility (ATTD, %) = [((nutrient in feed (g)× nutrient intake (g)) - (nutrient in feces (g)× fecal output (g)))/ (nutrient in feed (g) × feed intake (g)))] × 100

Total nutrient excretion (g) = (nutrient in feces (g) × fecal output (g)) + (nutrient in urine (g) × urinary output (g))

Nutrient retention (g) = nutrient intake (g) - total nutrient excretion (g)

Retention (% intake) = [nutrient retention (g)/ nutrient intake (g)] × 100

Retention (% digestible) = [nutrient retention (g)/ (nutrient intake (g) × ATTD coefficient (%))] × 100

The statistical models were implemented in SAS 9.4 (Statistical Analysis System, Cary, NC) using the GLIMMIX procedure, with pig as the experimental unit and the fixed effects of treatment, phase, and their interaction. Pig within block was treated as a repeated measure. Analysis of overall growth and nutrient balance of experimental phases 2 and 3 were analyzed by treatments. The UNIVARIATE procedure was used to verify normality and homoscedasticity of the Studentized residuals. Statistical outliers were identified as Studentized residuals greater than three standard deviations from the mean and were excluded from the analysis. Data were reported as least-square means and means separation was achieved using the probability of difference (PDIFF) option with Tukey adjustment for multiplicity. Results were considered significant if *P* ≤ 0.05 and a tendency if 0.05 < *P* ≤ 0.10 when no significant differences were detected across treatments (*P* > 0.05).

## Results

### General health

During the first sub-collection period for digestibility (days 9 to 12), two pigs exhibited symptoms of a health challenge (ie low feed intake, fever, lethargy). All pigs were then treated with 6 mL lincomycin (Lincomix 100^®^, Zoetis, Lincoln, NE) on days 9–11, respectively. These two pigs (one from NC and one from PC) were removed from the trial due to symptoms persisting for more than 24 h and interfering with proper sample collection. Due to subjects being removed prior to experimental diet administration, pigs were not included in statistical analysis. The second replicate of pigs were also administered with lincomycin on days 9–11.

### Diet analysis

Results of feed proximate analysis indicated that CP levels were slightly lower than expected across all treatments in both experimental phases; however, as planned, PC, WX, VP, WXVP, and SD diets were similar in CP, and NC was 6.5% lower (Tables 2 and 3). Total lysine levels showed some variation but were similar across formulated treatment values in all phases. Analyzed Ca values were higher than expected, resulting in a higher ratio of total Ca-to-total P (Ca: P); however, this was consistent across all treatments. Diets were formulated using a phytase additive with an expected enzyme activity of 2400 phytase units/g (FYT/g), and analyzed phytase activity was 2562 FYT/g. The source of xylanase that was used had an expected activity of 5000 xylanase units/g (FXU/g), and the analyzed activity was 6302 FXU/g. Beta glucanase that was used had an expected activity of 50 beta glucanase units/kg (FBG/kg), and analyzed values were 51 FBG/kg.

### Growth performance

Pigs started the experiment averaging 52.55 ± 4.12 kg and ended on day 36 at an average BW of 86.56 ± 5.03 kg. There was no evidence of dietary treatment differences for BW (*P* = 0.43; Table 4). Average daily gain (ADG), average daily feed intake (ADFI), and feed efficiency (G: F) were similar across treatments (*P* ≥ 0.13). There were no significant treatment effects on overall growth performance in pigs fed experimental treatments in phase 2 and 3 (*P* ≥ 0.57; Table 5).

**Table 4:** Effect of dietary treatment (NC, PC, WX, VP, WXVP, and SD) and collection period on pig body weight, average daily gain, average daily feed intake, and gain-to-feed ratio in growing pigs fed 22% (15.6%) or 41% (35%) soybean meal.

Item	Dietary treatment						SEM^g^	Treatment	Trt × Day
	NC^a^	PC^b^	WX^c^	VP^d^	WXVP^e^	SD^f^		*P*-value	*P*-value
**BW, kg**							0.690	0.43	0.70
** D0**	52.70	52.28	52.63	52.43	52.63	52.63			
** D12**	61.62	61.05	62.09	61.05	61.76	62.29			
** D24**	74.15	73.75	74.73	73.68	74.10	75.33			
** D36**	86.15	85.95	86.68	86.33	85.81	88.45			
**ADG, kg/d**							0.059	0.96	0.99
** Day 0–12**	0.76	0.74	0.79	0.74	0.76	0.81			
** Day 12–24**	1.06	1.07	1.05	1.08	1.05	1.09			
** Day 24–36**	1.01	1.03	1.00	1.04	1.00	1.02			
**ADFI, kg/d**							0.036	0.13	0.31
** Day 0–12**	1.36	1.33	1.37	1.35	1.37	1.39			
** Day 12–24**	1.53	1.48	1.53	1.50	1.55	1.54			
** Day 24–36**	1.71	1.70	1.75	1.70	1.76	1.71			
**G: F**							0.030	0.81	0.96
** Day 0–12**	0.55	0.55	0.57	0.54	0.56	0.58			
** Day 12–24**	0.69	0.72	0.69	0.71	0.67	0.71			
** Day 24–36**	0.59	0.60	0.58	0.63	0.57	0.60			

a NC; basal control diet formulated to represent a standard commercial diet with AA inclusions and 22% (15.6%) SBM.

b PC; high soybean meal diet (41%; 35%) formulated to replace all synthetic lysine.

c WX; high soybean meal diet (41%; 35%) with xylanase (Ronozyme WX 5000, DSM-Firmenich).

d VP; high soybean meal diet (41%; 35%) with inclusion of a multi-enzyme additive including hemicellulase and pectinase (Ronozyme VP, DSM- Firmenich).

e WXVP; high soybean meal diet (41%; 35%) with inclusion of xylanase (Ronozyme WX 5000, DSM-Firmenich) and a multi-enzyme additive including hemicellulase and pectinase (Ronozyme VP, DSM-Firmenich).

f SD; high soybean meal diet (41%; 35%) with 1500 phytase units (FYT) per kg of phytase (HiPhorious, DSM-Firmenich).

g SEM, standard error of the mean.

**Table 5:** Effect of dietary treatment (NC, PC, WX, VP, WXVP, and SD) on overall experimental collection (day 12–36) of ADG, ADFI, and G: F in growing pigs fed 22% (15.6%) or 41% (35%) soybean meal.

Item	Dietary Treatment						SEM^g^	Treatment	Treatment × Phase
	NC^a^	PC^b^	WX^c^	VP^d^	WXVP^e^	SD^f^		*P*-value	*P*-value
**ADG, kg**	1.03	1.04	1.03	1.07	1.02	1.05	0.029	0.85	0.99
**ADFI, kg**	1.62	1.60	1.63	1.58	1.66	1.63	0.035	0.57	0.31
**G: F**	0.64	0.66	0.64	0.67	0.62	0.65	0.023	0.65	0.96

a NC; basal control diet formulated to represent a standard commercial diet with AA inclusions and 22% (15.6%) SBM.

b PC; high soybean meal diet (41%; 35%) formulated to replace all synthetic lysine.

c WX; high soybean meal diet (41%; 35%) with xylanase (Ronozyme WX 5000, DSM-Firmenich).

d VP; high soybean meal diet (41%; 35%) with inclusion of a multi-enzyme additive including hemicellulase and pectinase (Ronozyme VP, DSM- Firmenich).

e WXVP; high soybean meal diet (41%; 35%) with inclusion of xylanase (Ronozyme WX 5000, DSM-Firmenich) and a multi-enzyme additive including hemicellulase and pectinase (Ronozyme VP, DSM-Firmenich).

f SD; high soybean meal diet (41%; 35%) with 1500 phytase units (FYT) per kg of phytase (HiPhorious, DSM-Firmenich).

g SEM, standard error of the mean.

### Apparent total tract digestibility

There were no significant differences across treatments for apparent total tract digestibility (ATTD) of DM and GE (*P* ≥ 0.22; Table 6). Overall ash digestibility was higher in pigs fed WX (67.45%), VP (70.14%), WXVP (67.80%), SD (69.01%) and tended to be higher in PC (66.77%) compared to NC (59.75%) (*P* < 0.01; Table 6). Ash digestibility was similar across HSBM (PC, WX, VP, WXVP, and SD) diets regardless of enzyme inclusion.

**Table 6:** Effect of dietary treatment (NC, PC, WX, VP, WXVP, and SD) on overall experimental collection (day 12–36) of apparent total tract digestibility (dry matter basis) of growing pigs fed 22% (15.6%) or 41% (35%) soybean meal.

Item, %	Dietary treatment						SEM^11^	Treatment	Treatment × Phase
	NC^5^	PC^6^	WX^7^	VP^8^	WXVP^9^	SD^10^		*P*-value	*P*-value
**Dry Matter^1^**	89.33	88.08	90.04	90.51	90.13	89.88	0.509	0.34	0.67
**Ash^2^**	59.75^a,b^^*#^	66.77^a,b^^*#^	67.45^a,b^	70.14^a,b^	67.80^a,b^	69.01^a,b^	1.837	< 0.01	0.08
**GE^3^**	88.69	88.77	89.78	90.29	89.77	89.61	0.538	0.22	0.65

1 Dry Matter, dry matter apparent total tract digestibility.

2 Ash, ash apparent total tract digestibility.

3 GE; gross energy apparent total tract digestibility.

4 NC; basal control diet formulated to represent a standard commercial diet with AA inclusions and 22% (15.6%) SBM.

5 PC; high soybean meal diet (41%; 35%) formulated to replace all synthetic lysine.

6 WX; high soybean meal diet (41%; 35%) with xylanase (Ronozyme WX 5000, DSM-Firmenich).

7 VP; high soybean meal diet (41%; 35%) with inclusion of a multi-enzyme additive including hemicellulase and pectinase (Ronozyme VP, DSM- Firmenich).

8 WXVP; high soybean meal diet (41%; 35%) with inclusion of xylanase (Ronozyme WX 5000, DSM-Firmenich) and a multi-enzyme additive including hemicellulase and pectinase (Ronozyme VP, DSM-Firmenich).

9 SD; high soybean meal diet (41%; 35%) with 1500 phytase units (FYT) per kg of phytase (HiPhorious, DSM-Firmenich).

10 SEM, standard error of the mean.

a,b Treatment means without a common superscript differ (*P* ≤ 0.05).

*# Treatment means without a common superscript tend to differ (0.05 ≤ *P* ≤ 0.10).

### Nitrogen balance

Nitrogen intake was higher in the experimental phase diets containing HSBM compared to the NC diet, with intake levels of 51.24–53.52 g/d and 35.60 g/d, respectively (*P* < 0.01; Table 7). Fecal N excretion levels were similar across treatments (*P* = 0.91). Urinary outputs were greater in WX, VP, and WXVP diets, with outputs being 10.14, 12.22, and 10.00 g/d greater than the NC, respectively (*P* < 0.01). Total N excretion was also greater in WX, VP, and WXVP diets, with total outputs being 10.03, 11.82, and 9.83 g/d greater than the NC, respectively (*P* < 0.01). Digestible N was greater in the HSBM diets compared to the NC (*P* < 0.01), digestibility levels were similar across HSBM diets regardless of enzyme inclusion. Nitrogen retention expressed on an absolute basis (g/d) differed among treatments (*P* = 0.02). Pigs fed the PC and SD diets exhibited the greatest N retention, whereas pigs fed the NC diet hat the lowest N retention. Intermediate values were observed for pigs fed WX, VP, and WXVP, which did not differ significantly from PC or SD. There were also no differences across treatments for N retention as a percent of intake or digestibility coefficients (*P* ≥ 0.78).

**Table 7:** Effect of dietary treatment (NC, PC, WX, VP, WXVP, and SD) on overall experimental collection (day 12–36) of nitrogen balance (dry matter basis) of growing pigs fed 22% (15.6%) or 41% (35%) soybean meal.

Item	Dietary treatment						SEM^7^	Treatment	Treatment × Phase
	NC^1^	PC^2^	WX^3^	VP^4^	WXVP^5^	SD^6^		*P*-value	*P*-value
**N intake, g d^−1^**	35.60^a^	51.42^b^	52.82^b^	52.07^b^	53.52^b^	52.20^b^	0.628	< 0.01	< 0.01
**Fecal N, g d^−1^**	4.79	4.71	4.68	4.39	4.62	4.77	0.276	0.91	0.77
**Urine N, g d^−1^**	19.23^a^	25.57^ab^	29.37^b^	31.45^b^	29.23^b^	26.55^ab^	2.443	< 0.01	0.06
**Total N excretion, g d^−1^**	24.02^a^	30.28^ab^	34.05^b^	35.84^b^	33.85^b^	31.32^ab^	2.453	0.01	0.21
**Digestible N, g d^−1^**	30.81^a^	46.71^b^	48.14^b^	47.68^b^	48.90^b^	47.43^b^	0.689	< 0.01	< 0.01
**N retention, g d^−1^**	11.47^a^^*^	21.02^b^	18.77^ab^	19.33^b^^#^	19.67^b^^#^	20.88^b^	2.133	0.02	0.10
**N retention, % of N intake**	33.02	40.74	35.65	36.91	36.95	40.19	4.247	0.79	0.88
**N retention, % of dig. N**	36.21	44.72	39.13	40.34	40.49	44.17	4.679	0.78	0.84

1 NC; basal control diet formulated to represent a standard commercial diet with AA inclusions and 22% (15.6%) SBM.

2 PC; high soybean meal diet (41%; 35%) formulated to replace all synthetic lysine.

3 WX; high soybean meal diet (41%; 35%) with xylanase (Ronozyme WX 5000, DSM-Firmenich).

4 VP; high soybean meal diet (41%; 35%) with inclusion of a multi-enzyme additive including hemicellulase and pectinase (Ronozyme VP, DSM- Firmenich).

5 WXVP; high soybean meal diet (41%; 35%) with inclusion of xylanase (Ronozyme WX 5000, DSM-Firmenich) and a multi-enzyme additive including hemicellulase and pectinase (Ronozyme VP, DSM-Firmenich).

6 SD; high soybean meal diet (41%; 35%) with 1500 phytase units (FYT) per kg of phytase (HiPhorious, DSM-Firmenich).

7 SEM, standard error of the mean.

a,b Treatment means without a common superscript differ (*P* ≤ 0.05).

*# Treatment means without a common superscript tend to differ (0.05 ≤ *P* ≤ 0.10).

### Phosphorus, calcium, and sulfur balance

Phosphorus intake was higher in diets containing HSBM (PC, WX, VP, WXVP, and SD) with 7.16–7.49 g/d compared to the NC diet with 6.20 g/d (*P* < 0.01; Table 8). Fecal, urine, and total P outputs were similar across treatments (*P* ≥ 0.10). Digestible P was higher in HSBM diets consisting of 4.31–4.81 g/d, compared to the NC with 3.57 g/d of digested P (*P* < 0.01). Absolute P retention was also higher in pigs fed PC, WX, VP, and SD diets compared to the NC treatment, 4.02–4.57 g/d vs. 3.47 g/d of retained P, respectively intermediate levels were observed in WXVP (*P* < 0.01). There were no observed differences across treatments for retention as a percent of intake or digestible P coefficients (*P* ≥ 0.26). Intake, excretion levels, digestible P, and retention metrics were similar across HSBM (PC, WX, VP, WXVP, and SD) diets regardless of enzyme inclusion.

**Table 8:** Effect of dietary treatment (NC, PC, WX, VP, WXVP, and SD) on overall experimental collection (day 12–36) of phosphorus balance (dry matter basis) of growing pigs fed 22% (15.6%) or 41% (35%) soybean meal.

Item	Dietary treatment						SEM^7^	Treatment	Treatment × Phase
	NC^1^	PC^2^	WX^3^	VP^4^	WXVP^5^	SD^6^		*P*-value	*P*-value
**Intake, g d^−1^**	6.20^a^	7.38^b^	7.49^b^	7.41^b^	7.28^b^	7.16^b^	0.100	< 0.01	< 0.01
**Fecal, g d^−1^**	2.63	2.74	2.97	2.61	2.97	2.49	0.155	0.10	0.47
**Urine, g d^−1^**	0.10	0.19	0.21	0.24	0.28	0.28	0.058	0.19	0.38
**Total P excretion, g d^−1^**	2.73	2.93	3.17	2.84	3.25	2.77	0.178	0.16	0.56
**Digestible P**	3.57^a^	4.64^b^	4.52^b^	4.81^b^	4.31^b^	4.67^b^	0.173	< 0.01	< 0.01
**Retention, g d^−1^**	3.47^a^	4.45^b^	4.32^b^	4.57^b^	4.02^ab^	4.38^b^	0.198	< 0.01	< 0.01
**Retention, % of intake**	55.78	60.20	57.71	61.59	55.64	61.20	2.472	0.26	0.34
**Retention, % of dig. P**	97.13	95.78	95.35	94.75	93.19	93.66	1.398	0.35	0.52

1 NC; basal control diet formulated to represent a standard commercial diet with AA inclusions and 22% (15.6%) SBM.

2 PC; high soybean meal diet (41%; 35%) formulated to replace all synthetic lysine.

3 WX; high soybean meal diet (41%; 35%) with xylanase (Ronozyme WX 5000, DSM-Firmenich).

4 VP; high soybean meal diet (41%; 35%) with inclusion of a multi-enzyme additive including hemicellulase and pectinase (Ronozyme VP, DSM- Firmenich).

5 WXVP; high soybean meal diet (41%; 35%) with inclusion of xylanase (Ronozyme WX 5000, DSM-Firmenich) and a multi-enzyme additive including hemicellulase and pectinase (Ronozyme VP, DSM-Firmenich).

6 SD; high soybean meal diet (41%; 35%) with 1500 phytase units (FYT) per kg of phytase (HiPhorious, DSM-Firmenich).

7 SEM, standard error of the mean.

a,b Treatment means without a common superscript differ (*P* ≤ 0.05).

Intake levels of Ca were highest in PC, WXVP, and SD; intermediate in WX and VP; and lowest in NC treatments (*P* < 0.01; Table 9). Fecal Ca outputs tended to be higher in pigs fed WXVP (3.51 g/d) compared to PC (2.76 g/d) and VP (2.80 g/d) (*P* = 0.07). Urinary Ca outputs tended to be lower in the SD treatment (0.28 g/d) compared to the PC treatment (0.64 g/d) (*P* = 0.05). Overall Ca excretion was higher in pigs fed WXVP (4.01 g/d) compared to the VP treatment (3.15 g/d) (*P* = 0.03). Digestible Ca was highest in the PC and SD treatments with 9.18 and 8.54 g/d, intermediate in the WX, VP, and WXVP treatments with 7.86, 7.85, and 7.96 g/d, respectively, and lowest in the NC with 6.73 g/d (*P* < 0.01). Absolute Ca retention levels were highest in PC and SD, intermediate in WX, VP, and WXVP and lowest in the NC treatments (*P* < 0.01). Retention as a percent of intake tended to be higher in PC and SD compared to the NC (*P* = 0.06). Retention as a percent of digestible Ca was higher in SD with 96.77% compared to the NC with 91.92% (*P* = 0.05). Total Ca excretion, retention as a percentage of intake and digestible Ca were similar across HSBM (PC, WX, VP, WXVP, and SD) diets regardless of enzyme inclusion.

**Table 9:** Effect of dietary treatment (NC, PC, WX, VP, WXVP, and SD) on overall experimental treatment collection (day 12–36) of calcium balance (dry matter basis) of growing pigs fed 22% (15.6%) or 41% (35%) soybean meal.

Item	Dietary treatment						SEM^7^	Treatment	Treatment × Phase
	NC^1^	PC^2^	WX^3^	VP^4^	WXVP^5^	SD^6^		*P*-value	*P*-value
**Intake, g d^−1^**	9.66^a^	11.94^c^	11.31^bc^	10.65^b^	11.46^c^	11.58^c^	0.199	< 0.01	< 0.01
**Fecal, g d^−1^**	2.93	2.76*	3.45	2.80*	3.51^#^	3.04	0.211	0.07	0.20
**Urine, g d^−1^**	0.55	0.64*	0.41	0.35	0.50	0.28^#^	0.093	0.05	0.05
**Total Ca excretion, g d^−1^**	3.48^ab^	3.41^ab^	3.85 ^ab^	3.15^a^	4.01^b^	3.31 ^ab^	0.219	0.03	0.07
**Digestible Ca, g d^−1^**	6.73^a^	9.18^c^	7.86^b^	7.85^b^	7.96^b^	8.54^bc^	0.255	< 0.01	< 0.01
**Retention, g d^−1^**	6.18^a^	8.53^c^^*^	7.46^b^	7.51^b^^#^	7.46^b^	8.27^c^	0.262	< 0.01	< 0.01
**Retention, % of intake Ca**	64.27*	71.41^#^	65.82	70.60	65.43	71.58^#^	1.918	0.06	< 0.01
**Retention, % of dig. Ca**	91.92^a^	93.16	94.43	95.55	93.85	96.77^b^	1.187	0.05	< 0.01

1 NC; basal control diet formulated to represent a standard commercial diet with AA inclusions and 22% (15.6%) SBM.

2 PC; high soybean meal diet (41%; 35%) formulated to replace all synthetic lysine.

3 WX; high soybean meal diet (41%; 35%) with xylanase (Ronozyme WX 5000, DSM-Firmenich).

4 VP; high soybean meal diet (41%; 35%) with inclusion of a multi-enzyme additive including hemicellulase and pectinase (Ronozyme VP, DSM- Firmenich).

5 WXVP; high soybean meal diet (41%; 35%) with inclusion of xylanase (Ronozyme WX 5000, DSM-Firmenich) and a multi-enzyme additive including hemicellulase and pectinase (Ronozyme VP, DSM-Firmenich).

6 SD; high soybean meal diet (41%; 35%) with 1500 phytase units (FYT) per kg of phytase (HiPhorious, DSM-Firmenich).

7 SEM; standard error of the mean.

a–c Treatment means without a common superscript differ (*P* ≤ 0.05).

*# Treatment or period means without a common superscript tend to differ (0.05 ≤ *P* ≤ 0.10).

Sulfur intake was higher in diets containing HSBM (PC, 3.48; WX, 3.58; VP, 3.58; WXVP, 3.58; SD, 3.52 g/d) compared to the NC diet with 2.72 g/d (*P* < 0.01; Table 10). Fecal and total S excretion was similar across treatments (*P* ≥ 0.12). A tendency of greater urinary S was observed in WXVP compared to NC, with 1.74 g/d and 1.35 g/d, respectively (*P* = 0.09). Digestible S was greater in HSBM treatments (PC, WX, VP, WXVP) compared to the NC (*P* < 0.01). Absolute retention values were greater in PC, WX, VP, and SD treatments compared to the NC (*P* ≤ 0.02); intermediate levels were observed in WXVP. However, there were no differences across treatments for retention of intake or digestible S coefficients (*P* ≥ 0.57). Intake, excretion levels, digestible S, and retention metrics were similar across HSBM (PC, WX, VP, WXVP, and SD) diets regardless of enzyme inclusion.

**Table 10:** Effect of dietary treatment (NC, PC, WX, VP, WXVP, and SD) on overall experimental collection (day 12–36) of sulfur balance (dry matter basis) of growing pigs fed 22% (15.6%) or 41% (35%) soybean meal.

Item	Dietary treatment						SEM^7^	Treatment	Treatment × Phase
	NC^1^	PC^2^	WX^3^	VP^4^	WXVP^5^	SD^6^		*P*-value	*P*-value
**Intake, g d^−1^**	2.72^a^	3.48^b^	3.58^b^	3.58^b^	3.58^b^	3.52^b^	0.035	< 0.01	< 0.01
**Fecal, g d^−1^**	0.60	0.57	0.60	0.55	0.61	0.62	0.031	0.52	0.63
**Urine, g d^−1^**	1.35*	1.54	1.66	1.66	1.74^#^	1.61	0.098	0.09	0.24
**Total S excretion, g d^−1^**	1.95	2.11	2.26	2.21	2.35	2.22	0.105	0.12	0.30
**Digestible S, g d^−1^**	2.12^a^	2.91^b^	2.98^b^	3.03^b^	2.97^b^	2.91^b^	0.046	< 0.01	< 0.01
**Retention, g d^−1^**	0.87^a^	1.37^b^	1.33^b^	1.36^b^	1.23^ab^	1.30^b^	0.113	0.02	0.06
**Retention, % of intake**	32.04	39.20	37.06	38.20	34.44	36.91	3.187	0.57	0.52
**Retention, % of dig. S**	40.81	46.82	44.67	45.06	41.30	44.61	3.693	0.79	0.69

1 NC; basal control diet formulated to represent a standard commercial diet with AA inclusions and 22% (15.6%) SBM.

2 PC; high soybean meal (41%; 35%) diet formulated to replace all synthetic lysine.

3 WX; high soybean meal diet (41%; 35%) with xylanase (Ronozyme WX 5000, DSM-Firmenich).

4 VP; high soybean meal diet (41%; 35%) with inclusion of a multi-enzyme additive including hemicellulase and pectinase (Ronozyme VP, DSM- Firmenich).

5 WXVP; high soybean meal diet (41%; 35%) with inclusion of xylanase (Ronozyme WX 5000, DSM-Firmenich) and a multi-enzyme additive including hemicellulase and pectinase (Ronozyme VP, DSM-Firmenich).

6 SD; high soybean meal diet (41%; 35%) with 1500 phytase units (FYT) per kg of phytase (HiPhorious, DSM-Firmenich).

7 SEM, standard error of the mean.

a,b Treatment means without a common superscript differ (*P* ≤ 0.05).

*# Treatment means without a common superscript tend to differ (0.05 ≤ *P* ≤ 0.10).

### Energy value and efficiency

Gross energy intake, digestible and metabolizable energy efficiencies, including ME: DE were similar across treatments (*P* ≥ 0.22; Table 11), indicating comparable energy utilization among diets.

**Table 11:** Effect of dietary treatment (NC, PC, WX, VP, WXVP, and SD) on overall experimental collection (day 12–36) of energy balance (dry matter basis) of growing pigs fed 22% (15.6%) or 41% (35%) soybean meal.

Item	Dietary treatment						SEM^i^	Treatment	Treatment × Phase
	NC^c^	PC^d^	WX^e^	VP^f^	WXVP^g^	SD^h^		*P*-value	*P*-value
**GE intake, Mcal d^−1^**	6.16	6.19	6.33	6.16	6.47	6.31	0.125	0.35	0.24
**DE^a^, %**	88.67	88.77	89.78	90.29	89.77	89.61	0.538	0.22	0.65
**ME^b^, %**	82.05	81.64	81.98	81.97	82.22	82.43	0.754	0.98	0.68
**ME/DE efficiency, %^c^**	92.52	91.95	91.32	90.79	91.59	91.58	0.612	0.39	0.47

a DE; percent digestible energy.

b ME; percent metabolizable energy.

c NC; basal control diet formulated to represent a standard commercial diet with AA inclusions and 22% (15.6%) SBM.

d PC; high soybean meal diet (41%; 35%) formulated to replace all synthetic lysine.

e WX; high soybean meal diet (41%; 35%) with xylanase (Ronozyme WX 5000, DSM-Firmenich).

f VP; high soybean meal diet (41%; 35%) with inclusion of a multi-enzyme additive including hemicellulase and pectinase (Ronozyme VP, DSM- Firmenich).

g WXVP; high soybean meal diet (41%; 35%) with inclusion of xylanase (Ronozyme WX 5000, DSM-Firmenich) and a multi-enzyme additive including hemicellulase and pectinase (Ronozyme VP, DSM-Firmenich).

h SD; high soybean meal diet (41%; 35%) with 1500 phytase units (FYT) per kg of phytase (HiPhorious, DSM-Firmenich).

i SEM, standard error of the mean.

## Discussion

In the present study, the authors hypothesized that including xylanase (WX), an enzyme complex of hemicellulase and pectinase (VP), a combination of both enzymes (WXVP), or higher inclusion of phytase (SD) in diets containing HSBM would help mitigate the antinutritional factors present in the diet. However, the result of this study showed no effects on growth performance and few improvements in nutrient digestibility metrics.

Although enzyme supplementation did not improve growth performance or markedly enhance nutrient digestibility, growth efficiency and lysine utilization were remarkable high across treatments. This suggests that pigs were already utilizing dietary AA with high efficiency, potentially limiting the opportunity for exogenous enzymes to provide additional performance responses or heightened levels of lysine. The experimental diets may have sufficiently met nutrient requirements despite the presence of antinutritional factors, therefore masking potential benefits of enzyme inclusion. Soybean meal quality parameters of phytate and trypsin inhibitor units were not analyzed, which may limit projections of these results to diets containing SBM of variable composition.

Neither xylanase, hemicellulase + pectinase, a combination of xylanase + hemicellulase + pectinase, or elevated phytase inclusion in diets containing HSBM altered ADG, ADFI, or G: F relative to the negative control (22.0% to 15.6% SBM corn-soy based diet) and positive control (41% to 35% SBM with and without out enzyme supplementation). Mechanisms which may explain the lack of differences across dietary treatments on growth performance metrics. Corn-soy based diets provide low digesta viscosity resulting in minimal breakdown of arabinoxylans and energy release (Navarro et al. 2018; Petry et al. 2020). Ward (2021) reported more consistent improvements in growth performance in response to xylanase in diets containing wheat and/or barley due to wheat and barley increasing the digesta viscosity and limiting nutrient absorption, with higher enzyme activity potential to act upon soluble NSPs present in these diets. Passos et al. (2015) have discussed the higher insolubility of xylans present in SBM, resulting in reduced enzyme activity to provide a fermentable substrate or influence an energy release contributing towards growth performance. Additionally, the AA-profile of SBM closely matches the AA requirements in pigs, and replacing crystalline AA with intact SBM elevates dietary CP but rarely limits growth unless an increased level of antinutritional factors are present (Woyengo et al. 2017). Even though dietary enzymes were active in the diet, their substrates contributed to no differences in energy release or change in AA supply, resulting in similar growth performance across all treatments. Interpretation of growth performance should account for the fixed feed allowance imposed in metabolism stalls, where intake was set at 2.8 times the maintenance energy requirement (197 kcal x BW^0.6^; NRC 2012) based on average pig BW, making BW response the primary variable of interest rather than voluntary feed intake.

Digestibility of DM, ash, and GE declined in phase 3, which has been reported previously in finishing pigs as body weight and gut capacity increase over time (Patience et al. 2015). In the present study, pigs were limit-fed a uniform amount twice daily, suggesting that the observed reduction in digestibility was more likely related to physiological maturity and changes in digesta passage rather than difference in feed intake among dietary treatments. Shorter digesta retention time in later growth stages may reduce substrate exposure to endogenous enzymes, resulting in lower apparent digestibility (Chassé et al. 2021). However, the prolonged period in metabolism stalls and restricted feeding to two time points per day may have also reduced digestibility measurements. Treatment differences were minimal due to the enzymes targeted substrates in the diet contributing towards a small, insoluble portion of the diet. Enzyme hydrolysis released limited additional digestible DM and GE, indicating comparable energy utilization among diets. The observed improved ash digestibility in the high SBM treatments compared to NC suggests that a higher presence of mineral-based ash in the HSBM diets improved ATTD compared to the NC (Debon and Tester 2001; Medic et al. 2014).

Increasing SBM from 22% to 41% in Phase 2 and 15.6% to 35% in Phase 3 raised dietary CP, N intake, and digestible N in HSBM treatments. Fecal N outputs were similar across treatments, but higher urinary and total N excretion were observed in WX, VP, and WXVP-fed pigs. An average increase of 5.76% in N excretion per 1% change in CP from the NC to the HSBM diets. This observation was slightly lower than the reported average N excretion being 8% per 1% CP fluctuation by Shurson and Kerr (2023). Millet et al. (2018) and van Milgen and Dourmad (2015) have highlighted that additional absorption of AA from the diet are deaminated and then excreted in the urine as urea after indispensable AA requirements are met, reflecting the higher urinary N excretions observed in the present study.

Absolute N retention was greater in HSBM diets compared to the NC diet. Comparing treatments, the PC, VP, WXVP, and SD had significantly higher N retention of 21.02, 19.33, 19.67, and 20.88 g/d, compared to the NC with 11.47 g/d. Greater absolute retention reflects a 38% increase in N intake on a grams per day basis and an average increase of 43% in digestible N present in higher SBM diets compared to the NC. Observed retention as a percent of intake and digestible N coefficients remained consistent across treatments. As intake, digestible N, and retention levels increase from NC to HSBM treatments, these retention estimates yielded similar values. Canh et al. (1997) has summarized that amino acids that are absorbed in the pig’s intestinal tract, but unutilized in the body after meeting their AA requirements, yields deamination of excess AA as ammonia and are converted to urea in the liver, resulting in elevated urea-N levels. The increase of urinary N excreted from pigs fed HSBM diets compared to the NC likely reflects heightened lysine present from greater SBM inclusion and increased release of AA from hydrolysis and later excreted, reflecting similar retention estimates as the remaining elevated SBM treatments (Ding et al. 2023).

Elevated Ca and P intake was observed in diets containing HSBM, however dietary enzyme inclusion and SBM inclusion had minimal effects on fecal and urinary excretion levels of Ca and P. The positive control diet (PC) and elevated phytase treatment (SD) improved Ca retention. Amerah et al. (2014) reported higher levels of Ca present in the diet and SBM can contribute towards Ca-phytate complexes, further preventing the bioavailability of P in the diet. However, Ravindran et al. (2008) have reported that phytase supplementation (≥500 FYT/kg) in broilers can improve the bioavailability of both Ca and P present in the diet. These results indicate that super-dosing phytase (SD) in HSBM diets was the only strategy that effectively digested and retained Ca, similar to the PC diet. Intermediate retention values observed in the WX, VP, and WXVP treatments may be due to enzyme hydrolyzation, but yielding substrates of NSP that re-bound Ca and limited its uptake (Selle et al. 2000; Sun et al. 2016).

When the phytase exceeds 1000 FYT kg^−1^ in the diet, additional cleavage sites are exposed deeper in the inositol ring, unlocking more minerals (Cowieson et al. 2006). Cao (2025) states that greater Ca: P ratios may decrease G: F and P digestibility due to Ca binding to phytate and decreasing the availability of available P, as previously mentioned, with further effects on feed efficiency. Cao (2025) observed decreased G: F in 50 kg BW pigs with a 1.55 Ca: P ratio in the absence of phytase, but with the same Ca: P ratio with diets containing 800 FYT/kg, there was an improved feed efficiency present from a feed efficiency of 0.37 to 0.39, respectively. Regardless of SBM inclusion, digestible and retained P did not differ, however, greater P retention observed in PC, WX, VP, and SD relative to NC suggests that increasing SBM or supplementation of dietary enzymes enhanced phosphorous utilization. No dietary effects on G: F were observed. The lack of treatment differences in HSBM treatments containing dietary enzymes may be due to similar Ca: P levels, however, intermediate retention observed for WXVP may indicate overlapping or non-additive effects of combined carbohydrase supplementation, potentially influenced by altered Ca: P ratios, similar to PC, WX, VP, and SD treatments and the NC.

Hydrolysis of insoluble xylans can yield arabinoxylan oligosaccharides that chelate Ca and Zn, forming complexes that are insoluble at intestinal pH and cause an increase in mineral loss (Debon and Tester 2001; Patience et al. 2022), reflecting upon the numerically lower ash ATTD present in WX (67.45%) and WXVP (67.80%) compared to VP (70.14%) and SD (69.01%). Additionally, if NSPs are only partially hydrolyzed in corn-soy diets, the insoluble oligosaccharide substrates can increase digesta viscosity and reduce the benefit of cell-wall digestion (Choct 1997; Bedford and Schulze 1998; Passos et al. 2015).

Neither NSP-degrading enzymes nor phytase act on sulfur-containing substrates (Choct 1997). Primary sulfur-containing substrates in the diet are found in sulfur-containing AA, methionine, and cysteine. Sulfur intake was elevated in diets containing HSBM, regardless of dietary enzyme inclusion, due to higher levels of methionine and cysteine present in SBM compared to corn (NRC 2012). Digestible S increased, reflective of increased intake present in higher SBM diets. However, absolute S retention and retention as a percent of intake and S digestibility coefficients remained unchanged across treatments. Excess absorbed sulfur-containing AA are trans-sulphurated and oxidized into sulfate, then retained mainly in the body (Hou et al. 2003; Stipanuk 2004).

As intake, digestible S, and retention levels increase from the NC to HSBM treatments, the retention estimates as a percent of intake and digestible S yielded similar values. Although intake and digestibility were higher in all HSBM treatments, there was no observed increase in S excretion levels. Absolute retention levels were comparable to digestibility metrics, which could be why there were similar S excretion levels due to higher retained S from the diet. Excess absorbed sulfur-containing AA are trans-sulphurated and oxidized into sulfate, then largely retained in the body (Hou et al. 2003; Stipanuk 2004).

No differences were observed in DE, ME, or ME: DE ratios across treatments. As previously discussed, corn-soy based diets have low levels of soluble NSP present in the diet, with > 90% of NSPs in both corn and SBM being insoluble, resulting in carbohydrase-mediated viscosity reduction yielding minimal additional fermentable carbohydrate (Bach-Knudsen 1997; Kocher et al. 2002). It has been reported that a slight increase in hindgut fermentation energy is offset by the heat increment of fermentation and the contribution of volatile fatty acids to net energy in growing pigs (Jørgensen et al. 1996), which may reflect comparable energy utilization among diets.

Elevating SBM to meet the lysine requirement in 53 kg pigs yielded no direct impact on growth performance but did increase N excretion. The present study showed a 5.27% increase in N excretion per 1% CP increase, also reflecting upon higher lysine in the HSBM treatments. Super-dose phytase effectively improves Ca and P utilization but was dependent on dietary Ca: P balance. Carbohydrase efficacy is observed to be diet specific. Carbohydrase activity yielded similar results to the positive control diet. Benefits of carbohydrases have yielded more consistent results in wheat and barley-based diets due to more soluble NSP present, and lower levels in corn-soy based diets due to higher insoluble NSP composition. Future research should investigate the effects of high-SBM with wheat or barley to supply substrates for xylanase, titrate dietary Ca: P in higher SBM diets with phytase, or quantify the further effects of greater N, S and P excretions on environmental impact from feeding elevated SBM.

## Abbreviations

SBM—: Soybean Meal
NC—: Negative Control, Moderate Soybean Meal Diet
PC—: Positive Control, High Soybean Meal Diet
WX—: High Soybean Meal Diet with Xylanase
VP—: High Soybean Meal Diet with Enzyme Complex, hemicellulase and pectinase
WX+VP—: High Soybean Meal Diet with Xylanase and Enzyme Complex
SD—: High Soybean Meal Diet with Superdose level of Phytase
ANF—: Antinutritional Factor
NSP—: Non-starch Polysaccharide
N—: Nitrogen
S—: Sulfur
Ca—: Calcium
P—: Phosphorous
Zn—: Zinc
NH_3—_: Ammonia
CH_4_ -: Methane
CP—: Crude Protein
AA—: Amino Acid
BW—: Body Weight
ADG—: Average Daily Gain
ADFI—: Average Daily Feed Intake
G: F—: Gain-to-feed efficiency
Orts—: Remaining Feed
ATTD—: Apparent Total Tract Digestibility
DM—: Dry Matter
OM—: Organic Matter
GE—: Gross Energy
DE—: Digestible Energy
ME—: Metabolizable Energy
ME: DE—: Metabolizable to Digestible Energy Efficiency

## References

[txag070-B1] Adeola O , Bedford M. R. 2004. Exogenous dietary xylanase ameliorates viscosity-induced anti-nutritional effects in wheat-based diets for white pekin ducks (*Anas platyrhynchos* domesticus). Br. J. Nutr. 92:87–94. 10.1079/BJN20041180 15230991

[txag070-B2] Amerah A. M. , Plumstead P. W., Barnard L. P., Kumar A. 2014. Effect of calcium level and phytase addition on ileal phytate degradation and amino acid digestibility of broilers fed corn-based diets. Poult. Sci. 93:906–915. 10.3382/ps.2013-03465 24706968

[txag070-B3] AOAC. 2007. Official methods of analysis. 17th ed. Assoc. Off. Anal. Chem., Arlington, VA.

[txag070-B4] Bach-Knudsen K. E. B. 1997. Carbohydrate and lignin contents of plant materials used in animal feeding. Anim. Feed Sci. Technol. 67:319–338. 10.1016/S0377-8401(97)00009-6

[txag070-B5] Bach-Knudsen K. E. B. 2014. Fiber and nonstarch polysaccharide content and variation in common crops used in broiler diets. Poult. Sci. 93:2380–2393. 10.3382/ps.2014-03902 25012855

[txag070-B7] Bedford M. A. , Schulze H. 1998. Exogenous enzymes for pigs and poultry. Nutr. Res. Rev. 11:91–114. 10.1079/NRR19980007 19087461

[txag070-B8] Canh T. T. , Verstegen M. W. A., Aarnink A. J. A., Schrama J. W. 1997. Influence of dietary factors on nitrogen partitioning and composition of urine and feces of fattening pigs. J. Anim. Sci. 75:700–706. 10.2527/1997.753700x 9078486

[txag070-B9] Cao S. C. et al 2025. Effects of total calcium-to-total phosphorus ratio on the calcium and phosphorus digestibility and growth performance of growing pigs. Anim. Feed Sci. Technol. 320:116197. 10.1016/j.anifeedsci.2024.116197

[txag070-B10] Chassé É. , Guay F., Bach-Knudsen K. E., Zijlstra R. T., Létourneau-Montminy M. P. 2021. Toward precise nutrient value of feed in growing pigs: effect of meal size, frequency and dietary fibre on nutrient utilisation. Animals. 11:2598. 10.3390/ani11092598 34573564 PMC8471499

[txag070-B11] Choct M. 1997. Feed milling international. June Issue. p. 13–26.

[txag070-B12] Cowieson A. J. , Acamovic I., Bedford M. R. 2006. Phytic acid and phytase: implications for protein utilization by poultry. Poult. Sci. 85:878–885. 10.1093/ps/85.5.878 16673766

[txag070-B13] Cromwell G. L. , Coffey R. D., Monegue H. J., Randolph J. H. 1995. Efficacy of low-activity, microbial phytase in improving the bioavailability of phosphorus in corn-soybean meal diets for pigs. J. Anim. Sci. 73:449–456. 10.2527/1995.732449x 7601778

[txag070-B14] Debon S. J. , Tester R. F. 2001. In vitro binding of calcium, iron and zinc by non-starch polysaccharides. Food Chem. 73:401–410. 10.1016/S0308-8146(00)00312-5

[txag070-B15] Dersjant-Li Y. , Awati A., Schulze H., Partridge G. 2015. Phytase in non-ruminant animal nutrition: a critical review on phytase activities in the gastrointestinal tract and influencing factors. J. Sci. Food Agric. 95:878–896. 10.1002/jsfa.6998 25382707 PMC4368368

[txag070-B158930351] Ding J et al 2023. Direct synthesis of urea from carbon dioxide and ammonia. Nat. Commun. 14:4586. 10.1038/s41467-023-40351-5 37524739 PMC10390537

[txag070-B16] Hatfield J. 2002. Minimizing the environmental impact of the pig industry. Proc. Int. Pig Vet. Soc.

[txag070-B17] Hou C. , Wykes L. J., Hoffer L. J. 2003. Urinary sulfur excretion and the nitrogen/sulfur balance ratio reveal nonprotein sulfur amino acid retention in piglets. J. Nutr. 133:766–772. 10.1093/jn/133.3.766 12612150

[txag070-B18] Humer E. , Schwarz C., Schedle K. 2015. Phytate in pig and poultry nutrition. J. Anim. Physiol. Anim. Nutr. 99:605–625. 10.1111/jpn.12258 25405653

[txag070-B19] Jørgensen H. , Zhao X.-Q., Eggum B. O. 1996. The influence of dietary fibre and environmental temperature on the development of the gastrointestinal tract, digestibility, degree of fermentation in the hind-gut and energy metabolism in pigs. Br. J. Nutr. 75:365–378. 10.1079/BJN19960140 8785211

[txag070-B22] Kocher A. , Choct M., Porter M. D., Broz J. 2002. Effects of feed enzymes on nutritive value of soyabean meal fed to broilers. Br. Poult. Sci. 43:54–63. 10.1080/00071660120109890 12003339

[txag070-B23] Medic J. , Atkinson C., Hurburgh C. R. 2014. Current knowledge in soybean composition. J. Am. Oil Chem. Soc. 91:363–384. 10.1007/s11746-013-2407-9

[txag070-B24] Millet S et al 2018. Pork production with maximal nitrogen efficiency. Animal. 12:1060–1067. 10.1017/S1751731117002610 29065938

[txag070-B25] National Research Council. 2012. Nutrient requirements of swine. 11th rev. ed. Natl. Acad. Press, Washington, DC.

[txag070-B26] Navarro D. M. , Bruininx E. M., de Jong L., Stein H. H. 2018. Effects of physicochemical characteristics of feed ingredients on the apparent total tract digestibility of energy, DM, and nutrients by growing pigs. J. Anim. Sci. 96:2265–2277. 10.1093/jas/sky149 29688508 PMC6095346

[txag070-B27] Passos A. A. , Andrade C., Phillips C. E., Coffey M. T., Kim S. W. 2015. Nutrient value of spray field forages fed to pigs and the use of feed enzymes to enhance nutrient digestibility. J. Anim. Sci. 93:1721–1728. 10.2527/jas.2014-8435 26020194

[txag070-B28] Patience J. F. , Rossoni-Serão M. C., Gutiérrez N. A. 2015. A review of feed efficiency in swine: biology and application. J. Anim. Sci. Biotechnol. 6:33. 10.1186/s40104-015-0031-2 26251721 PMC4527244

[txag070-B29] Patience , J. F.Li, Q., Petry A. L. 2022. Xylanases and cellulases: relevance in monogastric nutrition pigs. In: M. R. Bedford, G. Partridge, C. L. Walk, M. Hruby, editors, Enzymes in farm animal nutrition. CABI, Wallingford, UK. p. 33–51. 10.1079/9781789241563.000

[txag070-B30] Petry A. L. , Huntley N. F., Bedford M. R., Patience J. F. 2020. Xylanase increased the energetic contribution of fiber and improved the oxidative status, gut barrier integrity, and growth performance of growing pigs fed insoluble corn-based fiber. J. Anim. Sci. 98:skaa233. 10.1093/jas/skaa233 32687554 PMC7392531

[txag070-B32] Ravindran V. , Cowieson A. J., Selle P. H. 2008. Influence of dietary electrolyte balance and microbial phytase on growth performance, nutrient utilization, and excreta quality of broiler chickens. Poult. Sci. 87:677–688. 10.3382/ps.2007-00247 18339988

[txag070-B34] Selle P. H. , Ravindran V., Caldwell A., Bryden W. L. 2000. Phytate and phytase: consequences for protein utilisation. Nutr. Res. Rev. 13:255–278. 10.1079/095442200108729098 19087442

[txag070-B35] Shurson G. C. , Kerr B. J. 2023. Challenges and opportunities for improving nitrogen utilization efficiency for more sustainable pork production. Front. Anim. Sci. 4:1204863. 10.3389/fanim.2023.1204863

[txag070-B36] Stipanuk M. H. 2004. Sulfur amino acid metabolism: pathways for production and removal of homocysteine and cysteine. Annu. Rev. Nutr. 24:539–577. 10.1146/annurev.nutr.24.012003.132418 15189131

[txag070-B37] Sun N et al 2016. Food protein-derived calcium chelating peptides: a review. Trends Food Sci. Technol. 58:140–148. 10.1016/j.tifs.2016.10.004

[txag070-B38] Swanstrom J. A. et al 2023. Effect of soybean meal inclusion on grow-finish pig performance and nitrogen balance. J. Anim. Sci. 101:251–252. 10.1093/jas/skad341.285

[txag070-B39] Torres-Pitarch A. , Manzanilla E. G., Gardiner G. E., O’Doherty J. V., Lawlor P. G. 2019. Systematic review and meta-analysis of the effect of feed enzymes on growth and nutrient digestibility in grow-finisher pigs: effect of enzyme type and cereal source. Anim. Feed Sci. Technol. 251:153–165. 10.1016/j.anifeedsci.2018.12.007

[txag070-B40] Trabue S. , Kerr B., Bearson B., Ziemer C. 2011. Swine odor analyzed by odor panels and chemical techniques. J. Environ. Qual. 40:1510–1520. 10.2134/jeq2010.0522 21869513

[txag070-B41] van Milgen J , Dourmad J. Y. 2015. Concept and application of ideal protein for pigs. J. Anim. Sci. Biotechnol. 6:15. 10.1186/s40104-015-0016-1 25937926 PMC4416387

[txag070-B42] Villca, B et al 2016. Effect of a carbohydrase enzyme complex on the nutrient apparent total tract digestibility of rye-based diets fed to growing-finishing pigs under liquid feeding. J. Anim. Sci. 94:230–233. 10.2527/jas.2015-9747

[txag070-B44] Wang Y et al 2018. Advances in low-protein diets for swine. J. Anim. Sci. Biotechnol. 9:60. 10.1186/s40104-018-0276-7 30034802 PMC6052556

[txag070-B45] Ward N. E. 2021. Debranching enzymes in corn/soybean meal–based poultry feeds: a review. Poult. Sci. 100:765–775. 10.1016/j.psj.2020.10.074 33518131 PMC7858153

[txag070-B46] Woyengo T. A. , Beltranena E., Zijlstra R. T.. 2017. Effect of anti-nutritional factors of oilseed co-products on feed intake of pigs and poultry. Anim. Feed Sci. Technol. 233:76–86. 10.1016/j.anifeedsci.2016.05.006

